# Supplementary material for: Tetrandrine alleviates cerebral ischemia/reperfusion injury by suppressing NLRP3 inflammasome activation via Sirt-1
Source: PeerJ. 2020 May 7;8:e9042. doi: 10.7717/peerj.9042 (PMC7211409; doi:10.7717/peerj.9042)
Supplement: Supplemental Information 6 [file peerj-08-9042-s006.pptx]

## Slide 1
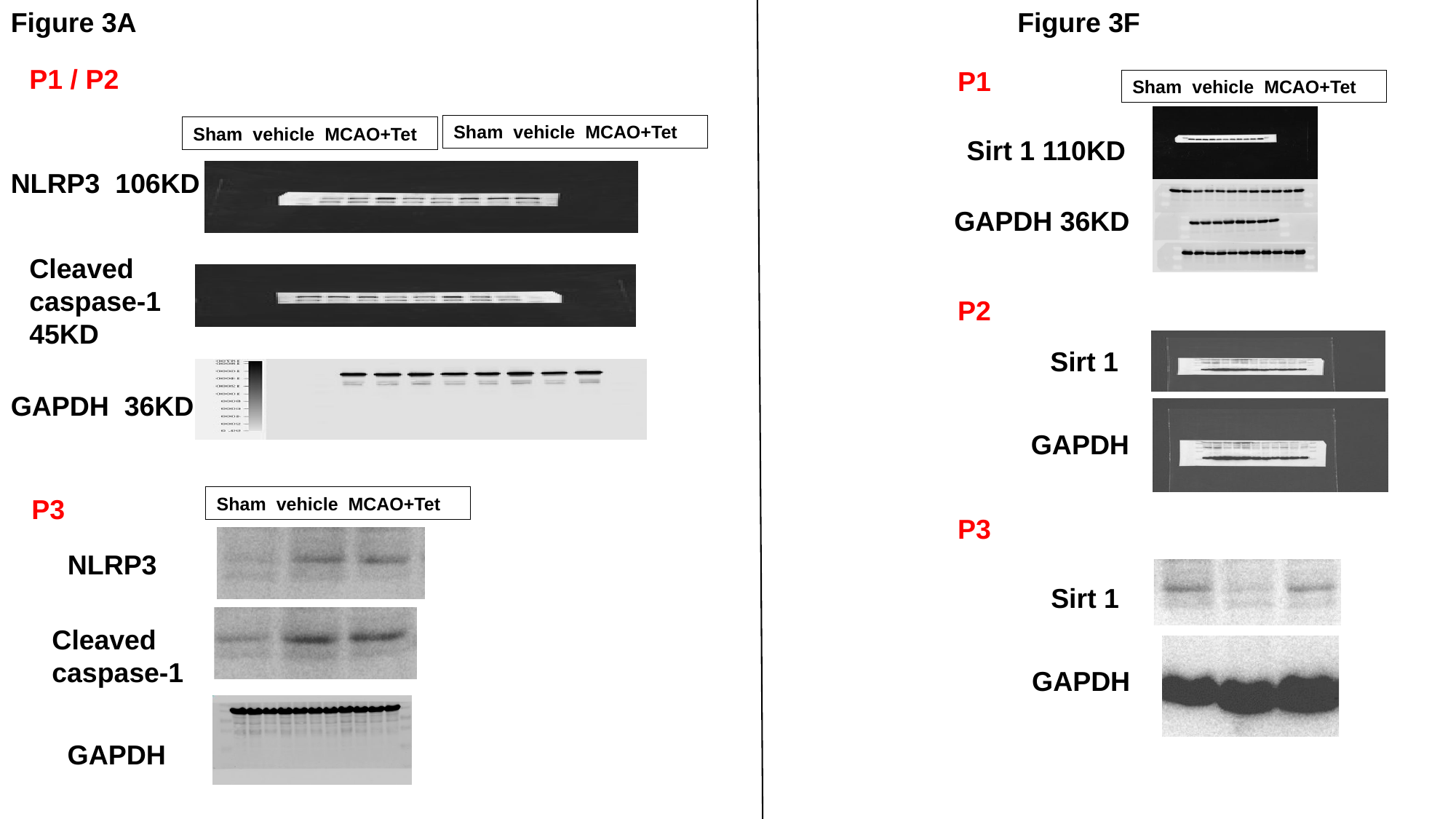

Figure 3A
Figure 3F
P1 / P2
P1
Sham vehicle MCAO+Tet
Sham vehicle MCAO+Tet
Sham vehicle MCAO+Tet
Sirt 1 110KD
NLRP3 106KD
GAPDH 36KD
Cleaved
caspase-1 45KD
P2
Sirt 1
GAPDH 36KD
GAPDH
P3
Sham vehicle MCAO+Tet
P3
NLRP3
Sirt 1
Cleaved
caspase-1
GAPDH
GAPDH

## Slide 2
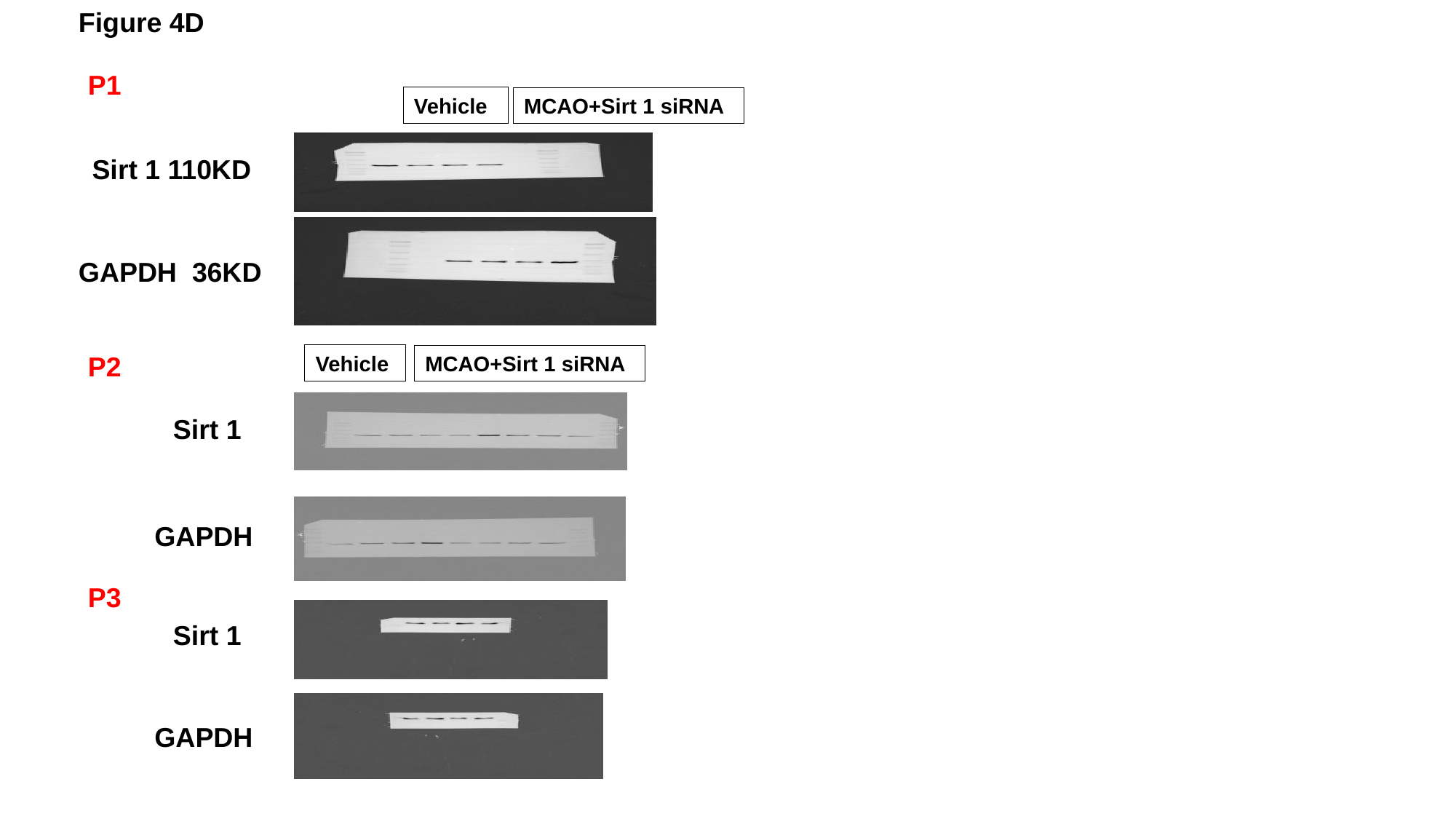

Figure 4D
P1
Vehicle
MCAO+Sirt 1 siRNA
Sirt 1 110KD
GAPDH 36KD
P2
Vehicle
MCAO+Sirt 1 siRNA
Sirt 1
GAPDH
P3
Sirt 1
GAPDH
